# A Third Emerging Stage for the Current Digital Society? Optimal Parenting Styles in Spain, the United States, Germany, and Brazil

**DOI:** 10.3390/ijerph16132333

**Published:** 2019-07-02

**Authors:** Fernando Garcia, Emilia Serra, Oscar F. Garcia, Isabel Martinez, Edie Cruise

**Affiliations:** 1 Department of Methodology of the Behavioral Sciences, University of Valencia, Av. Blasco Ibanez, 21, 46010 Valencia, Spain; 2 Department of Developmental and Educational Psychology, University of Valencia, Av. Blasco Ibanez, 21, 46010 Valencia, Spain; 3 Department of Psychology, University of Castilla-La Mancha, Avda de los Alfares 44, 16071 Cuenca, Spain; 4 Department of Economics and Social Work, University of Trier, Universitätsring 15, D-54296 Trier, Germany

**Keywords:** family socialization, parental warmth, parental strictness, parenting styles

## Abstract

We propose a new paradigm with three historical stages for an optimal parenting style (i.e., indulgent parenting style), which extends the traditional paradigm of only two stages (i.e., authoritarian and authoritative parenting styles). The three stages concur, at the same time, in different environments, context, and cultures. We studied the third stage for optimal parent–child relationships through the offspring’s personal and social well-being, with four adolescent samples from 11 to 19 years old (52.2% girls) from Spain (*n* = 689), the United States (*n* = 488), Germany (*n* = 606), and Brazil (*n* = 672). The offspring’s personal well-being was measured through self-esteem (academic, social, emotional, family, and physical), while social well-being was measured with the internalization of self-transcendence (universalism and benevolence) and conservation values (security, conformity, and tradition). The parent–child parenting style was measured through parental warmth and strictness, and the adolescents’ parents were classified into one of four groups (indulgent, authoritarian, authoritative, and neglectful). Remarkably, the greatest personal well-being was found for adolescents raised with higher parental warmth and lower parental strictness (i.e., indulgent), and the greatest social well-being was found for adolescents raised with higher parental warmth (i.e., indulgent and authoritative; *p* < 0.05 for all countries). Consistently, poorer personal well-being and social well-being were associated with less parental warmth (i.e., authoritarian and neglectful). Findings suggest that the parent–child relationships analyzed have a common pattern associated with personal and social well-being that coincide with a proposed third stage.

## 1. Introduction

Parents raise their children within a specific time and cultural environment. Parenting literature has traditionally suggested two different historical stages of optimal parenting styles over the past century or so. Early in the last century, in a first stage, for example, John B. Watson (1928) [1] warned parents about spoiling their children with superfluous displays of affection and warmth, while recommending strictness—imposing regular habits on them in order to instill self-discipline, following an authoritarian style. In the historical second stage, considering an industrial society perspective and unclear parenting research evidence, Laurence Steinberg (2001) [2] strengthened the idea that parental warmth and parental strictness, characterizing the authoritative style, are both key to children’s well-being in “contemporary, industrialized societies” (Steinberg, 2001, p. 13) [2]. Furthermore, the current emergent research in the digital era is beginning to seriously doubt whether the parental strictness and imposition component of certain parenting styles is still needed in order to foster the personal and social well-being of adolescents [3,4,5]. In this work, we posit that a third stage perspective is needed in order to fully understand an optimal parenting style in the current digital era.

### 1.1. The Past Century Paradigm with Two Parenting Stages Perspectives

Traditionally, numerous studies have captured parent–child relationships in two main orthogonal dimensions—identified as warmth and strictness (Darling and Steinberg, 1993, pp. 491–492 [6]; Smetana, 1995, p. 299 [7]; Steinberg, 2005, p. 71 [8]) or labels with similar meaning [9]. The parental dimension of warmth describes the degree to which parents demonstrate their care and acceptance to their children, and how they support and communicate with them. The warmth dimension has been labeled with other names with a similar meaning, such as responsiveness, assurance, implication, or involvement. The dimension of parental strictness refers to the degree parents establish the norms for their children’s behavior. This dimension has traditionally been labeled with other names, such as demandingness, domination, hostility, inflexibility, control, restriction, or parental firmness [4,6,8,10,11]. Four parenting styles have been derived from these dimensions—authoritative (characterized by both warmth and strictness), authoritarian (characterized by strictness but lacking warmth), indulgent (characterized by warmth without strictness), and neglectful (lacking both warmth and strictness) [4,10,12]. A parenting-styles approach captures the overarching, persisting parenting characteristics; better integrates and organizes particular parenting practices; and accurately organizes the relationships among parenting styles, parenting practices, and their associations with children’s personal and social well-being [4,6,8,10,12,13,14,15].

Since the early 1900’s, numerous studies have repeatedly verified that the authoritative parenting style (both warmth and strictness) is optimal for children and adolescents. Authoritativeness during childhood has been clearly and repeatedly associated with good functioning, even in late adulthood. Authoritative parenting was identified as optimal (the highest parent–child relationship quality) for children and adolescents from middle-class European–American families [12,16,17]. Even beyond adolescence, authoritativeness in childhood has been associated with positive functioning in late adulthood [18,19]. Warmth and strictness (which define the authoritative parenting style) have both been found to be critical to children’s development [16,20,21,22,23]. Authoritative parents would offer emotional support by means of warmth (acceptance and involvement), and would establish adequate guidelines and limits to control children’s behavior through strictness [2,16]. Because of the diversity of the cultural values present in these and other studies conducted, Steinberg (2001) [2] came to note that the benefits of authoritative parenting cut across the boundaries of ethnic background, socioeconomic status, and household structure, from an industrialized society perspective.

Furthermore, classical studies have also widely recognized that the authoritarian parenting style (strictness lacking warmth) leads to optimal adjustment, in ethnic minorities in the United States [24,25], hierarchical collectivistic countries [26,27], and sociocultural environments where the implications of disobeying parental rules may be of grave and detrimental consequence to the self and others [28,29,30]. Even the earliest literature on parenting supports the idea that the parenting style that is normative in one culture may not be normative in another. Some studies found differences among black and white youth concerning the authoritarian parenting style, specifically in youth outcomes, such as cognitive competence, social competence, and lower internalizing problems, where there were positive associations for black youth, but not for their white counterparts (e.g., Brody and Flor, 1998 [31]). Baumrind (1972) [24] analyzed the differences in race by parenting style, in addition to the preschooler behavior effects from the parenting style, in her landmark study. She found that black children raised under the authoritarian style showed better outcomes, compared with white children, which could indicate a difference in what scoring highly on authoritarian parenting means [28].

### 1.2. The Three Parenting Stages Perspectives

Different but related lines of argumentation have been suggested in order to explain these variations in the universality of the authoritative parenting style being optimal. Framed within the person–environment fit model, according to the ideas of the ecology of human development (Bronfenbrenner, 1986 [32]), studies have suggested that people fit better in environments where their attitudes, values, and experiences are held in common. As low socioeconomic status families of ethnic minorities are more likely to live in hazardous communities where crime is higher, authoritarian parenting may not be as harmful in this environment, and it may even have some protective benefits [33]. In agreement with the first stage that characterizes the initial studies of parenting in the beginning of the century, some societies and cultural contexts seem persistently related to the authoritarian parenting style as being optimal [28]. For example, authoritarian parenting practices in black communities are seen as caring, loving, respectful, protective, and beneficial for the child [34]. Moreover, in an environment where disobedience may result in harm to the self and others, an authoritarian parenting style could possibly be as functional as other styles [28,29].

Horizontal and vertical individualism and collectivism macrosocial concepts have been used by researchers to explain the observed differences in the relation between parenting styles and child adjustment [35,36,37], whereby studies carried out in Asian and Arab societies show that children in these collectivist cultures understand the individual self as part of the family self. In such societies, the expectation is for intergenerational relationships to be vertical and hierarchical, with strictness and imposition representing a major component of parental responsibility. Strict authoritarian discipline is viewed as being in children’s best interest, while if such discipline were lacking, it would be viewed as an absence of supervision and care [26,38]. Conversely, studies carried out mainly in Spain and Brazil have suggested that in horizontal collectivist cultures, such as South American or some European countries, the self is also conceptualized as part of a larger group (the family), but in contrast to hierarchical cultures, the organization of the group is egalitarian, rather than hierarchical [4,39,40]. Horizontal collectivist cultures underscore egalitarian relations, and the use of affection, acceptance, and involvement in raising children is of greater focus. Additionally, strictness and firm control in child rearing seem to be perceived negatively in horizontal collectivist cultures [4,35,39]. Recent emerging studies continuously reinforce this perspective, analyzing Spanish adolescents and older adults [41], traditional bullying and cyberbullying victimization [42,43], reactive and proactive adolescent violence [44], child-to-parent violence [45,46], parenting children with poor school performance [47], antisocial tendencies [48,49], and drug-use problems [3,50].

However, beyond the clear nationwide limits, recent evidence seems to indicate that traditional vertical individualist societies (i.e., Great Britain) and horizontal individualist societies (i.e., Sweden) are moving toward a third stage, where an indulgent parenting style seems to be optimal. Strictness practices do not seem to be effective, and high levels of reasoning, parental affection, acceptance, and involvement would be enough to obtain optimal adolescent adjustment (even for drug-use, e.g., [3,4,5]), without needing the authoritative component of high-levels of strictness. A study conducted with a large sample of European adolescents (Sweden, Slovenia, Czech Republic, the United Kingdom, Spain, and Portugal) found that regardless of the country, an authoritative parenting style and an indulgent parenting style (support without strictness and imposition to set limits) were equally protective against drug-use, but the indulgent parenting style performed even better than the authoritative parenting style when examining the outcomes of self-esteem and school performance. This pattern persisted across the sample set, even among adolescents from two archetypal individualist countries in Northern Europe (i.e., the United Kingdom [3] and Sweden [5]). Furthermore, in analyzing parenting styles beyond adolescence, a recent study with samples in Great Britain found that high-care is beneficial for well-being, self-esteem, and social competence, regardless of the level of strictness, with a common pattern in both the short- and long-term (from adolescence to early older age) [51]. Additionally, recent meta-analyses examining the relations between parenting styles with externalizing problems [52,53], behavior problems, and academic achievement [54], and self-esteem in children and adolescents [55], are starting to recognize the benefits of indulgent parenting. These emergent findings suggest the need for a third stage, with a new perspective on the family, in contrast to the previous perspective on the family, where both parental warmth and parental strictness were key to children’s well-being. In this new third stage, parental strictness and imposition seem not only not beneficial, but even harmful, and so the parental warmth dimension is enough to support children when they behave well, and to correct children’s misconduct through reasoning and communicative practices [4,40,56].

Finally, the relation of parenting styles with those patterns of adjustment and maladjustment have shown to be consistent across adolescent age and sex, despite the multiple differences that have been established in different aspects of adolescent adjustment depending on age and sex. For example, it has been confirmed that girls tend to present higher academic self-esteem, whereas boys tend to have higher emotional and physical self-esteem [39,40,50]. In the same way, adolescents tend to score higher than older adults in some self-esteem dimensions, such as social and family self-esteem [42,50], especially early adolescents, who have shown higher family, emotional, and physical self-esteem than older adolescents [50]. Contrastingly, values internalization tends to be higher in older adults than in adolescents [42].

### 1.3. The Present Study

This study aims to examine the parent–child relationship quality, and the positive personal and social well-being outcomes of adolescents from four countries. We test the third stage paradigm with data from Spain (horizontal-collective culture), the United States (vertical-individualist culture), Germany (horizontal-individualist culture), and Brazil (horizontal-collective culture) [39,40,57,58]. 

The positive personal well-being of the offspring was captured through multidimensional self-esteem (academic, social, emotional, family, and physical), while the social well-being of the offspring was captured through the internalization of self-transcendence values (universalism and benevolence) and conservation values (security, conformity, and tradition). Both the child’s self-esteem and the internalization of social values are central objectives of parental socialization [59].

Self-esteem has been one of the traditional outcomes of children’s adjustment in parenting studies [35], and one of the main keys to positive personal well-being [50,60,61,62], which captures more than only self-discipline [1]. Different authors have repeatedly stressed the importance of parenting styles in children’s internalization of social values [35,38,56]. Internalization, defined as, “taking over the values and attitudes of society as one’s own so that socially acceptable behavior is motivated not by anticipation of external consequences but by intrinsic or internal factors” (Grusec and Goodnow, 1994, p. 4 [59]), has been established as a key distinctive component of positive well-adjusted children [6,21,22,63]. This internalization of social values can only be fully articulated in a parental context of parental warmth, responsiveness, and involvement shared by authoritative and indulgent parenting styles. This said internalization even emphasizes positive effects on others, fostering a child’s feelings of empathy and consideration for others [22,64]. Self-transcendence and conservation values focus on consideration for others and acceptance of social norms, becoming goals that guide adult development [65,66,67].

In this study, we investigate the positive development of children, considering that well-being is not limited to the absence of behavioral disorders (e.g., drug-use of adolescents). Any socialization context (that transforms individuals into social human beings) should always have a self-discipline component, but also preserve, or even develop, the individual self of the child as part of the person. The internalization of social values guarantees the quality of the socialization process, by not only getting children to obey the social norms [39,40,66], but also by internalizing them. Parents are the main source of influence for children’s well-being, and they can enable a positive self (high self-esteem) in their child [21]. Positive self-esteem is a main aim of positive parenting, and, by extension, by positive psychology.

Based on the literature review, we hypothesize a third stage. We expect that high levels of parental warmth (present in both the authoritative and indulgent parenting styles) will be associated with better socialization outcomes (self-esteem and internalization of values) among adolescents from four countries. We expect this association will be consistent, independent of the sex and age of the participants.

## 2. Materials and Methods

### 2.1. Participants

The sample was composed of 2455 students (52.2% women) covering the adolescent age range (aged 11 to 19 years old, mean (M) = 15.24, standard deviation (SD) = 1.98)—1350 early (55.0%, from 11 to 15 years old) and 1105 late (45.0%, from 16 to 19 years old) adolescents. Sampled from Spain (689, 28.1%; 50.4% being women; mean age = 14.53, SD = 1.77, range = 11–18 years; 455, 66.0%, being early adolescents), United States (488, 19.9%; 49.0% being women; mean age = 15.61, SD = 1.29, range = 13–19 years; 249, 51.0%, being early adolescents), Germany (606, 24.7%; 58.3% being women; mean age = 16.07, SD = 2.12, range = 12–17 years; 250, 41.3%, being early adolescents), and Brazil (672, 27.4%; 51.0% being women; mean age = 14.95, SD = 2.14, range = 11–17 years; 396, 58.9%, being early adolescents).

### 2.2. Procedure

The sample frame of the present study was adolescents from secondary schools from large metropolitan areas (with over one million inhabitants in each area) on the East Coast Spain, the Midwestern United States, Middle West Germany, and in the Northeast of Brazil. The data was collected from 26 educational centers (six Spanish, five North American, seven German, and eight Brazilian), selected through the simple random sampling method from a complete list of centers [4,42,62,68,69]. In the samples of the four countries, we selected adolescents from middle class neighborhoods who (a) lived in two-parent nuclear families, with a mother or primary female caregiver and father or primary male caregiver, and (b) their parents and four grandparents were born in the country of each sample (Spain, Germany, Brazil, and the United States) [4,70]. Additionally, in the case of the sample of the United States, we only selected white European–American adolescents [4,25,70].

An a priori power analysis was computed so as to calculate the minimum sample size that was required in order to fix the conventional statistical errors of type I (α = 0.05) and type II (β = 0.05) when fixing a medium–small effect size (*f* = 0.17, estimated from ANOVAs of Lamborn et al., 1991 [12]) in a univariate *F*-test between the four parenting style groups [71,72]. The a priori power analyses (α = 0.05; 1 – β = 0.80; *f* = 0.17) showed a minimum sample size of 384 participants. In the four countries, the sample size was always over what was planned. A post-hoc power analysis [71,72] showed that the *F*-probe could detect in the worst case (the United States: *n* = 488; α = 0.05; β = 0.20) the expected effect size (*f* = 0.17), with a power that exceeded the a priori fixed value (1 − β = 0.90). On the other hand, the sensitivity power analysis with the full sample (*n* = 2455; α = β = 0.05) indicated that the *F* main effects between the four parenting styles could detect even a small effect size (*f* = 0.08) [71,72,73].

We obtained the approval to carry out this study through the Valencian Research Ethics Committee of the Program for the Promotion of Scientific Research, Technological Development, and Innovation in Spain. Next, the research was approved in the Research and Evaluation Boards of each city where the study was conducted. After that, the head or principal of each educational center gave their approval to conduct the study in the individual secondary schools. Finally, each teacher or instructor gave permission for the questionnaires to be completed during their class time. Our teams sent a letter to inform each student and their parents or legal guardians of the details of our questionnaires, as well as the purpose of our research. All of the participants had signed parental/guardian permission, and we also had the signed assent from the students themselves, assuring voluntary participation. All of the questionnaires were completed anonymously. We tested the questionnaires for aberrant response patterns, such as reporting implausible inconsistencies between negatively and positively worded responses or “maximum-scale” behavior [11,49,74,75,76,77]. Approximately 6% (*n* = 147) of the data set contained aberrant response patterns, and were removed from the sample.

### 2.3. Instruments

#### 2.3.1. Parental Socialization

Parental socialization was measured with the Parental Socialization Scale ESPA29 [78]. It is a self-report instrument designed to examine parenting styles through children’s and adolescents’ (aged 10 to 18 years) responses. The acceptance/involvement dimension was measured with warmth, reasoning, indifference, and detachment subscales (both the detachment and indifference subscales have a negative relation to the dimension). The following subscales measured the strictness/imposition dimension: revoking privileges, verbal scolding, and physical punishment. All of the subscales were measured in response to 29 situations that reflect the context of day-to-day family life between adolescents and their parents. There were 13 scenarios where the context of obedience was established, which is that the family norm is followed (e.g., “If I do what he/she tells me to do”), and 16 scenarios where the context was of disobedience, meaning that the family norm is broken (e.g., “If I break or ruin something at home”). The parenting practices of warmth (“He/she shows affection”) and indifference (“He/she seems indifferent) were measured in response to the 13 contexts of obedience, while the parenting practices of reasoning (“He/she talks to me”), detachment (“It’s the same to him/her”), verbal scolding (“He/she scolds me”), physical punishment (“He/she hits me”), and revoking privileges (“He/she takes something away from me”) were measured in response to the 16 disobedience contexts. A four-point scale was used to indicate how often the respondent’s mother and father employ the seven specified parenting practices, with ranges from one, meaning “never”; two, meaning “sometimes”; three, meaning “most times”; to four, meaning “always”.

The ESPA29 factor structure was confirmed with exploratory [9,78,79] and confirmatory [11,15] analyses. The instrument was originally developed and validated in Spain [78], and was also validated in the English [15], Portuguese [11], Brazilian-Portuguese [9,79], and Basque [80] languages. The ESPA29 dimensions and subscales have been applied to analyze multiple socialization outcomes, such as school adjustment [81], drug use [81,82], behavioral problems [83], neighborhood violence [70], reactive and proactive adolescent violence [44], bullying and cyberbullying [42], child-to-parent violence [45], self-concept [84], and prosocial values [40]. The Cronbach’s alpha, in the present study, for the two main dimensions, were the following: acceptance/involvement (0.968) and strictness/imposition (0.964). For each subscale, the Cronbach’s alpha values were warmth (0.961), indifference (0.950), reasoning (0.950), detachment (0.920), verbal scolding (0.954), physical punishment, 0.936, and revoking privileges (0.952).

#### 2.3.2. Multidimensional Self-Concept

The AF5 [85] questionnaire was designed to measure self-concept with the following five dimensions: academic (e.g., “I am a good student”), social (e.g., reversed item, “It is difficult for me to make friends”), emotional (e.g., reversed item, “I get scared easily”), family (e.g., “My parents give me a lot of confidence”), and physical (e.g., “I am an attractive person”). The scale consists of a total of 30 items across five dimensions of self-esteem, which are evenly distributed with six items measuring each dimension. The participant rates the statements according to his/her level of agreement or disagreement using a 99-point scale (portrayed by a thermometer), ranging from 1 = complete disagreement, to 99 = complete agreement. Modifications were made to obtain a score index ranging from 0.10 to 9.99.

The five-factor multidimensional structure of the AF5 was confirmed with exploratory [85] and confirmatory [74,86] analyses, and no method effect appears to be associated with negatively-worded items [76,77,85]. The instrument was originally developed and validated in Spain [85], and was also validated in the English [87], Portuguese [88], Brazilian-Portuguese [74], Basque [89], and Catalan [90] languages. The AF5 scales have been applied in multiple research fields, such as in connection with nature [91], academic performance [92], interpersonal communication [91,93], transcultural parenting [74], parenting with antisocial children [49] and adolescents with school problems [47], intergenerational parenting socialization [41], and parenting socialization in the current digital age [42]. The alpha reliability coefficients in the present study were as follows: academic (0.859), social (0.676), emotional (0.735), family (0.784), and physical (0.727).

#### 2.3.3. Internalization of Social Values

The social values internalization was measured with 27 items from the Schwartz (1992) [94] Value Inventory [39,40,41,66,95]. Self-transcendence higher order values included universalism (e.g., “Being at one with nature (integration with nature)”) and benevolence (e.g., “Faithful (loyal to my friends and to people I identify with)”) values subscales, and conservation higher order values included tradition (e.g., “Being accepting of life (assimilating the circumstances of life)”), conformity (e.g., “Courtesy (education and good manners)”), and security (e.g., “Reciprocity of favors (not being in debt with anyone)”) values subscales. The participant rated the items with a 99-point rating scale (portrayed by a thermometer), which ranges from 1 (opposed to my values) to 99 (of supreme importance). Modifications were made to obtain a score index ranging from 0.10 to 9.99. The conservation and self-transcendence higher order values are characterized as being oriented to social focus [66,95]. Conservation and self-transcendence values have been used in parenting research as child social outcomes [39,40,41]. The Schwartz Value Inventory scales have been used in hundreds of research areas, as varied as drug use [96] and abuse [97,98], or as the main key for underlying and undermining well-being across different countries [66]. Cronbach’s alphas for the subscales in present study were as follows: universalism (0.745), benevolence (0.721), security (0.564), conformity (0.689), and tradition (0.582). These reliability indices were within the range of variation commonly observed for these value types [39,40,66].

### 2.4. Data Analysis

To analyze the influence of parenting styles on socialization outcomes, a four-way multifactorial (4 × 4 × 2 × 2) multivariate analysis of variance (MANOVA) was applied to two sets of outcome variables (self-esteem and internalization of values) with parenting styles (authoritative, authoritarian, indulgent, and neglectful), country (Spain, the United States, Germany, and Brazil), age groups (early vs late adolescents), and sex (men vs women) as independent variables. Follow-up univariate *F*-tests were conducted for the outcome variables that had multivariate significant overall differences, and significant results on the univariate tests were followed up with Bonferroni comparisons of all possible pairs of means [4,12,17,62,68].

## 3. Results

### 3.1. Parenting Style Groups

Participants from the four countries (i.e., Spain, Brazil, the United States, and Germany) were classified into one of four parenting households (i.e., indulgent, authoritative, authoritarian, or neglectful; Table 1). The indulgent family contained 572 adolescents (23.3%) with high warmth, M = 3.47 and SD = 0.25, but low strictness, M = 1.37 and SD = 0.21; the authoritative family contained 659 (26.8%) with high warmth, M = 3.49 and SD = 0.45, and high strictness, M = 1.88 and SD = 0.25; the authoritarian group contained 574 (23.4%) with low warmth, M = 2.79 and SD = 0.31, and high strictness, M = 1.87 and SD = 0.33; and the neglectful family contained 650 (26.5%) with low warmth, M = 2.78 and SD = 0.32, and low strictness, M = 1.35 and SD = 0.21.

### 3.2. Preliminary Multivariate Analysis for Multidimensional Self-Esteem

The results for the MANOVA conducted in the five multidimensional self-esteem outcomes (i.e., academic, social, emotional, family, and physical) yielded significant main effects for the parenting style (Λ = 0.860, *F*(15, 6589.9) = 24.72, *p* < 0.001), sex (Λ = 0.875, *F*(5, 2387.0) = 68.37, *p* < 0.001), age (Λ = 0.989, *F*(5, 2387.0) = 5.26, *p* < 0.001), and country (Λ = 0.856, *F*(15, 6589.9) = 25.55, *p* < 0.001; Table 2). Additionally, interaction effects between sex and country (Λ = 0.981, *F*(15, 6589.9) = 3.13, *p* < 0.001), and age and country (Λ = 0.976, *F*(15, 6589.9) = 3.90, *p* < 0.001) were found.

### 3.3. Parenting Styles and Self-Esteem

The univariate results showed that parenting styles had statistically significant main effects in all self-esteem dimensions (see Table 2). Overall, indulgent parenting was related to equal or even better self-esteem than authoritative parenting; contrastingly, authoritarian and neglectful parenting were related to poor self-esteem. Regarding academic self-esteem, adolescents from indulgent homes obtained better scores than those from authoritative, authoritarian, and neglectful homes. Adolescents raised with authoritative parenting scored between those with indulgent parents (who reported the highest scores) and those with authoritarian and neglectful parents (who reported the lowest scores). For social self-esteem, adolescents from indulgent and authoritative households reported higher scores than their peers from authoritarian and neglectful families. Concerning emotional self-esteem, indulgent and neglectful parenting were related to higher scores than the authoritative and authoritarian styles. With respect to family self-esteem, adolescents from indulgent households reported higher scores than those with authoritative, authoritarian, and neglectful parents; authoritative parenting was associated with higher scores than authoritarian and neglectful parenting, and the lowest scores corresponded with authoritarian parenting. Finally, for physical self-esteem, the adolescents who characterized their parents as indulgent reported the highest scores, whereas the lowest scores corresponded with those raised by neglectful and authoritarian parents; additionally, authoritative parenting was related with higher scores than authoritative style.

### 3.4. Demographic Variables and Self-Esteem

**Figure 1:**
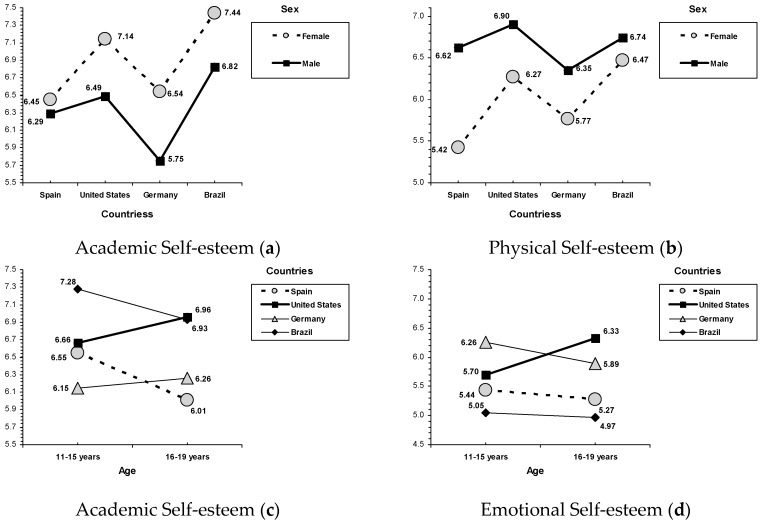

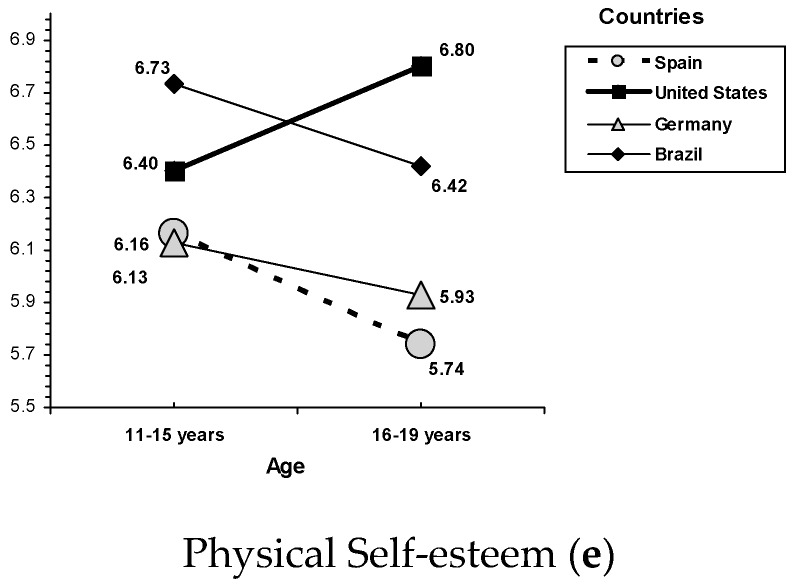
Interactions for sex and country. (**a**) Academic self-esteem and (**b**) physical self-esteem. Interactions for age and country. (**c**) Academic self-esteem, (**d**) emotional self-esteem, and (**e**) physical self-esteem.

Although not the focus of the present study, several univariate main effects for sex, age, and country attained a significant statistical level (see Table 3). The sex-related differences revealed that females reported more academic self-esteem, but less emotional and physical self-esteem than males. Additionally, an interaction between sex and country was found on academic self-esteem (*F*(3, 2391) = 3.64, *p* = 0.012), and physical self-esteem (*F*(3, 2391) = 8.57, *p* < 0.001; see Figure 1). In a similar way, although females reported higher academic self-esteem, this pattern was weaker in Spain than in the United States, Germany, and Brazil. Also, males have greater physical self-esteem than females, although this tendency was less clear in Brazil than in the other three countries. Age-related differences indicated that early adolescence (i.e., 11–15 years) was related to higher self-esteem than late adolescence (i.e., 16–19 years; see Table 3). Again, an interaction effect between age and country was found on academic self-esteem (*F*(3, 2391) = 9.08, *p* < 0.001), emotional self-esteem (*F*(3, 2391) = 6.15, *p* < 0.001), and physical self-esteem (*F*(3, 2391) = 4.78, *p* = 0.003; see Figure 1). Interestingly, age-related patterns in self-esteem outcomes showed a different trend by country. In the United States, late adolescents reported higher academic, emotional, and physical self-esteem than early adolescents. Opposingly, early adolescents from Spain and Brazil (in academic and physical self-esteem) and those from Germany (in emotional self-esteem) reported higher scores than their country-peers from the late adolescent group. Some country-related differences were found. Remarkably, on academic self-esteem, adolescents from the United States and Germany scored between the highest scores of Brazilian adolescents, and the lowest scores of Spanish and German adolescents. In contrast, on social self-esteem, the highest scores were reported by United States adolescents, the lowest by Brazilian adolescents, and adolescents from Spain and Germany were in the middle position. Finally, whereas Spanish and German adolescents reported the highest family self-esteem, the United States and Brazilian adolescents showed the highest physical self-esteem.

### 3.5. Preliminary Multivariate Analysis for Internalization of Social Values

The results for the MANOVA conducted in the social values of self-transcendence (i.e., universalism and benevolence) and conservation (i.e., security, conformity, and tradition) yielded significant main effects for parenting style (Λ = 0.933, *F*(15, 6589.9) = 11.16, *p* < 0.001), sex (Λ = 0.961, *F*(5, 2387.0) = 19.38, *p* < 0.001), age (Λ = 0.995, *F*(5, 2387.0) = 2.47, *p* = 0.031), and country (Λ = 0.796, *F*(15, 6589.9) = 37.89, *p* < 0.001). Additionally, the interaction effects between parenting style and age (Λ = 0.989, *F*(15, 6589.9) = 1.78, *p* = 0.031), parenting style and country (Λ = 0.966, *F*(45, 10,680.7) = 1.82, *p* < 0.001), age and country (Λ = 0.970, *F*(15, 6589.9) = 4.88, *p* < 0.001) were found.

### 3.6. Parenting Styles and Internalization of Social Values

Again, the results from the univariate analysis showed that adolescents who characterized their parents as indulgent and authoritative reported a greater priority to self-transcendence values (i.e., universalism and benevolence), as well as giving greater priority to conservation values (i.e., security, conformity, and tradition) than their peers who were raised by authoritarian and neglectful parents, whereas neglectful and authoritarian styles were constantly related to lower scores on all of the internalization of the values outcomes. Additionally, authoritarian parenting was associated with the poorest scores on priority to benevolence and conformity social values (see Table 4). 

**Figure 2:**
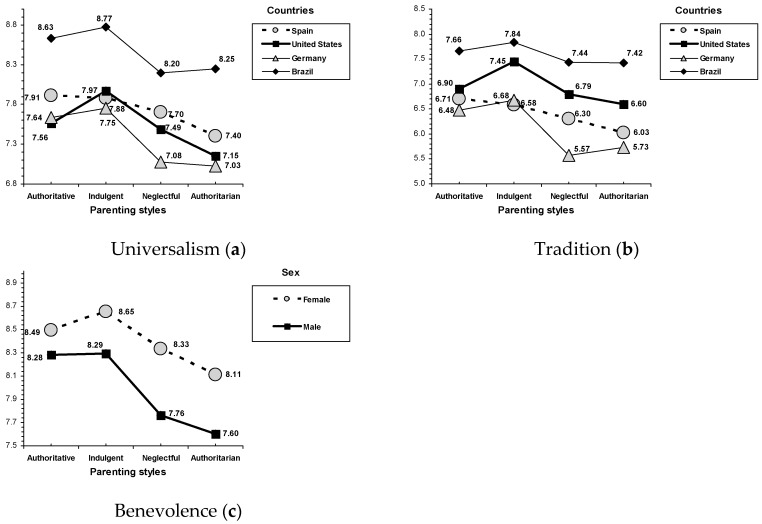
Interactions for parenting style by age: (**a**) universalism and (**b**) tradition. Interactions for parenting style by sex: (**c**) benevolence.

Furthermore, an interaction effect between parenting style and country was found on universalism (*F*(3, 2391) = 2.30, *p* = 0.015) and tradition (*F*(3, 2391) = 3.10, *p* = 0.001; see Figure 2). In a similar way, the parenting country profile revealed that adolescents from indulgent families gave equal or even higher priority to universalism and tradition (in the United States) than those adolescents raised by authoritative parents, whereas poor rates corresponded with adolescents who characterized their parents as authoritarian and neglectful (German adolescents with authoritarian and neglectful parents obtained the lowest scores). Additionally, an interaction effect between parenting style and sex was found on benevolence (*F*(3, 2391) = 3.30, *p* = 0.020; see Figure 2). Overall, despite females giving greater priority to benevolence than males, parenting sex profile revealed that, for males and females, indulgent and authoritative parenting were related with a higher priority to benevolence than authoritarian and neglectful parenting, although this tendency is greater in males.

### 3.7. Demographic Variables and Internalization of Social Values

**Figure 3:**
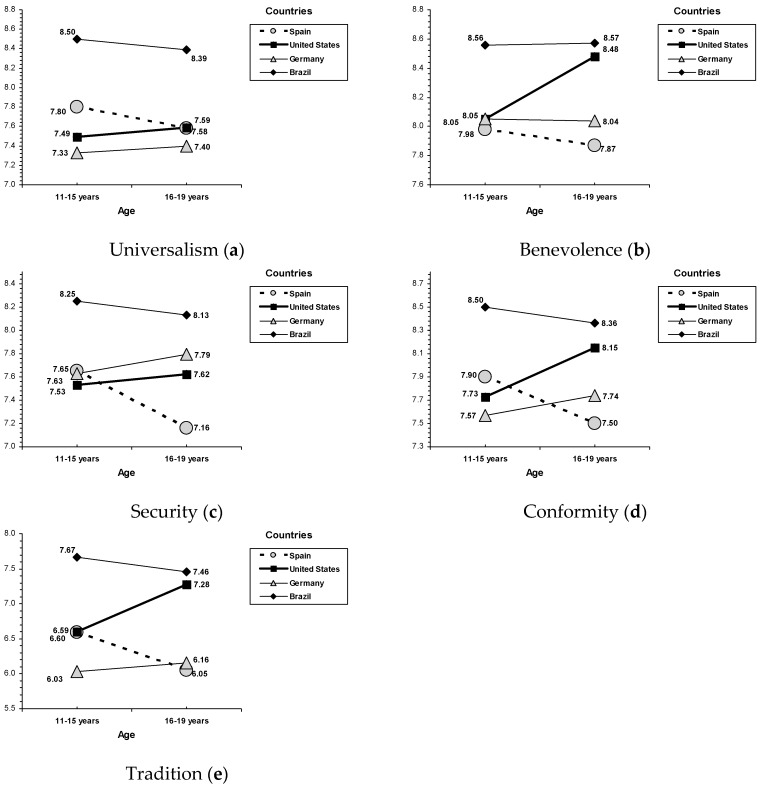
Interactions for age and country: (**a**) universalism, (**b**) benevolence, (**c**) security, (**d**) conformity, and (**e**) tradition.

The results from the univariate analysis applied showed that the univariate main effects for sex, age, and country reached a significant statistical level (see Table 3). The sex-related differences showed that females reported a higher priority to self-transcendence (i.e., universalism and benevolence) and conservation (security, conformity, and tradition) than males. Age-related differences showed a different profile for early adolescence (i.e., 11–15 years) and late adolescence (i.e., 16–19 years) as a function of country, and interaction effects between age and country were found on the self-transcendence values of universalism (*F*(3, 2391) = 2.91, *p* = 0.033) and benevolence (*F*(3, 2391) = 5.81, *p* = 0.001), and on conservation social of conformity (*F*(3, 2391) = 9.92, *p* < 0.001), tradition (*F*(3, 2391) = 16.28, *p* < 0.001), and security (*F*(3, 2391) = 7.87, *p* < 0.001; see Figure 3). In the United States, late adolescents (i.e., 16 to 19 years old) reported greater scores than early adolescents (i.e., 11 to 15 years old) in benevolence, conformity, and tradition; in Spain the highest scores corresponded with early adolescence (in security, conformity, and tradition); and few variations in social values between both age groups were found among Brazilian and German adolescents. Country-related differences examining the interactions between age and country revealed a general pattern—Brazilian adolescents reported the greatest scores, the lowest corresponded with adolescents from Spain and Germany, and North American adolescents were in the middle position. Interestingly, this country general tendency was different in late adolescence, in which those from Brazil and the United States obtained higher scores in benevolence, conformity, and tradition, whereas those from Spain and Germany reported lower scores.

## 4. Discussion

The present study examines the association between parenting styles with the social competence pattern and adjustment of Spanish, North American, German, and Brazilian adolescents from middle-class families through a two-dimensional four-typology model of parenting styles in a large sample. In order to capture social competence and adjustment among adolescents, we examined multidimensional self-esteem (i.e., academic, social, emotional, family, and physical), internalization self-transcendence social values (i.e., universalism and benevolence), and conservation social values (i.e., security, conformity, and tradition). Overall, our findings revealed that the indulgent parenting style was associated with optimal scores (highest self-esteem and internalization of social values) in Spain, the United States, Germany, and Brazil. In the four countries examined, adolescents from indulgent families obtained equal or even greater scores on well-being than those from authoritative households, whereas those from neglectful and authoritarian homes were consistently associated with poor levels of self-esteem and the internalization of social values.

Findings from the analysis examining the self-esteem outcomes revealed that parenting styles (i.e., indulgent, authoritative, authoritarian, and neglectful) and the five self-esteem indicators share a common pattern across the four countries examined. Interestingly, indulgent parenting was related with self-esteem equal to authoritative parenting in the social and physical domain. The indulgent style even overcame authoritative parenting in academic, emotional, and family self-esteem domains. In contrast, adolescents from authoritarian and neglectful families showed the poorest self-esteem. The results from the analysis examining the internalization of social values indicated that there were theoretically predictable differences in priority to self-transcendence (i.e., universalism and benevolence) and conservation (i.e., security, conformity, and tradition) among adolescents from the four family typologies. Adolescents from indulgent and authoritative families reported greater priority to both self-transcendence and conservation social values than their peers from authoritarian and neglectful homes. Additionally, the parenting country profile for universalism and tradition social values indicated that indulgent parenting was related to an equal or even greater internalization of social values than authoritative parenting (i.e., in the United States), whereas being raised by authoritarian and neglectful families was a risk factor for the internalization of social values (especially for German adolescents). In a similar way, the parenting profile for male and female adolescents in benevolence social values indicated that, despite females giving greater priority to benevolence than males, indulgent and authoritative parenting have a positive impact on the internalization of benevolence social values, whereas authoritarian and neglectful parenting were related to a poor priority for benevolence social values.

Furthermore, one important implication of this study for the literature on quality parenting and children’s wellbeing is that the combination of parental warmth and involvement, but not strictness and imposition, seems to be the best parenting strategy for the new third emergent stage in the current digital era, where the indulgent parenting style seems to be optimal. In sum, the warmth and involvement component of the parenting style underlies offspring’s well-being, whereas the strictness and imposition component undermines offspring’s well-being.

On the one hand, the results of this study have common implications that are also applied to the second stage of the socialization of industrialized societies where the optimal socialization style is authoritative [2]. The results of this research reinforce the idea that spontaneous disclosures of information to parents by their children (shared by authoritative and indulgent styles), but not the parents’ attempts to secure information (shared by authoritative and authoritarian styles), are strategic factors in the offspring’s well-being [3,20,23]. Accordingly, the offspring’s internalization of self-transcendence and conservation values involved socially-focused motivations, which the findings of this study clearly associated with indulgent and authoritative parenting styles [39,40,66], emphasizing the positive effects on others of fostering a child’s feelings of empathy and consideration for others [21,22,64]. Moreover, authoritarian and neglectful styles, both lacking the parenting component of warmth and involvement, share a lack of underlying social-focus [96,97,98] in their parenting, with implications of a lack of empathy and no consideration for others’ feelings [66,95]. 

On the other hand, in the third stage of socialization, the component of strictness and imposition (which is shared by authoritative and authoritarian) undermines the offspring of an authoritative parenting style. The indulgent parenting style was associated with the same (academic and physical self-esteem) or even higher personal adjustment (social, emotional, and family self-esteem) than the authoritative parenting style. These results for offspring’s personal and social well-being are different from the first and second stages. In the first stage, strictness is the only main parenting dimension that guarantees the offspring’s well-being [1,6,28,29,33]. In the same way, in the second stage, strictness is the main key, along with warmth and involvement, to fostering the offspring’s well-being [6,10,12,16,17].

Although one of the most important contributions of the present study is the common pattern between parenting styles, and competence and adjustment among adolescents from Spain, the United States, Brazil, and Germany, the results from the present study are in agreement with previous studies supporting the idea that adolescence could not be a homogenous life-time period for all cultures and countries [2,99]. In this sense, our results examining age-related differences in multidimensional self-esteem outcomes and the internalization of social values showed a different age-profile by country among early and late adolescents. In the United States, late adolescents reported better developmental outcomes than early adolescents on self-esteem (academic, emotional, and physical domains) and the internalization of social values (benevolence, conformity, and tradition). In contrast, early adolescence was associated with higher developmental outcomes than late adolescence in Spain (on academic and physical self-esteem, and the internalization of security, conformity, and tradition social values), Brazil (on academic and physical self-esteem), and Germany (on emotional self-esteem). Despite these age variations in adjustment and competence as a function of country, the findings of the present study conducted with middle-class adolescents from Spain, the United States, Brazil, and Germany suggest that indulgent parenting (i.e., warmth but not strictness) offers equal or even better results than authoritative parenting (warmth and strictness), in order to achieve two of the most important goals of parental socialization—developing adequate self-esteem as well as the internalization of social values.

Finally, this study has strengths and limitations. The use of the two-dimensional four-style model to assess parenting offers conceptual framework to the ongoing debates of parenting by examining parenting styles in a large context across different demographic variables, contexts, and countries. As for the limitations, the current study was cross-sectional, which does not allow us to draw firm conclusions about directionality. The classification of the families within one of the four parenting styles was based on the adolescent’s responses, although a common pattern of invariance was guaranteed [9,11,15]. 

## 5. Conclusions

Despite these limitations, the findings from this study reinforce the idea that considering the person’s fit to the context within a broader global context, using a three-stages conceptual framework that informs of the different co-existing relationships between parents’ socialization styles and their children’s well-being is needed. The different results found in parenting literature can be understood from this new three-stages perspective. Future research should also take the new third stage, proposed in this study, into account when outlining emerging positions in parenting literature. 

## Figures and Tables

**Table 1:** Number of cases in parenting style groups, mean scores, and standard deviations for main measures of parental dimensions. SD—standard deviation.

	Total	Authoritative	Indulgent	Authoritarian	Neglectful
Frequency	2445	659	572	574	650
Percent	100	26.8	23.3	23.4	26.5
Warmth					
Mean	3.15	3.49	3.47	2.79	2.78
SD	0.45	0.25	0.25	0.31	0.32
Strictness					
Mean	1.62	1.88	1.37	1.87	1.35
SD	0.38	0.32	0.21	0.33	0.21

**Table 2:** Means (and standard deviations) for parenting style, and the main univariate *F*-values for self-esteem and the internalization of social values (self-transcendence and conservation).

Self-Esteem	Parenting Style				
	Authoritative	Indulgent	Authoritarian	Neglectful	*F*(3, 2391)
Academic	6.82 ^2^	7.10 ^1^	6.20 ^3^	6.39 ^3^	28.85 ***
	(1.76)	(1.67)	(1.81)	(1.88)	
Social	7.47 ^1^	7.65 ^1^	7.10 ^2^	7.22 ^2^	14.88 ***
	(1.45)	(1.31)	(1.48)	(1.39)	
Emotional	5.30 ^2^	5.81 ^1^	5.28 ^2^	5.88 ^1^	16.51 ***
	(1.9)	(1.98)	(1.95)	(1.88)	
Family	8.43 ^2^	8.90 ^1^	7.37 ^4^	8.13 ^4^	100.01 ***
	(1.36)	(1.00)	(1.89)	(1.49)	
Physical	6.38 ^a^	6.60 ^1^	6.07 ^2,b^	6.12 ^2^	10.54 ***
	(1.91)	(1.83)	(1.82)	(1.81)	

* *p* < 0.05; ** *p* < 0.01; *** *p* < 0.001; Bonferroni test: α = 0.05; 1 > 2 > 3 > 4; a > b.

**Table 3:** Means (and standard deviations) for parenting style and school performance, and the main univariate *F*-values for the set of outcome measures (self-esteem and internalization of social values).

Outcome Measures	Sex			Age			Country				
	Female	Male	*F*(1, 2391)	11–15 Years	16–19 Years	*F*(1, 2391)	Spain	United States	Germany	Brazil	*F*(3, 2391)
**Self-esteem**											
Academic	6.87	6.36	63.248 ***	6.71	6.53	3.469	6.37 ^3^	6.81 ^2^	6.21 ^3^	7.14 ^1^	41.518 ***
	(1.78)	(1.82)		(1.88)	(1.73)		(1.85)	(1.83)	(1.73)	(1.70)	
Social	7.36	7.36	0.132	7.46	7.23	14.139 ***	7.43	7.43	7.27	7.31	1.533
	(1.50)	(1.34)		(1.39)	(1.46)		(1.35)	(1.43)	(1.52)	(1.41)	
Emotional	5.19	5.98	112.775 ***	5.52	5.62	0.158	5.38 ^2^	6.01 ^1^	6.04 ^1^	5.01 ^3^	47.424 ***
	(1.99)	(1.80)		(1.95)	(1.94)		(1.94)	(1.88)	(1.87)	(1.89)	
Family	8.22	8.20	0.231	8.30	8.11	13.873 ***	8.28 ^1^	8.04 ^2^	8.48 ^1^	8.03 ^2^	13.459 ***
	(1.64)	(1.48)		(1.53)	(1.59)		(1.52)	(1.62)	(1.34)	(1.70)	
Physical	5.96	6.66	77.378 ***	6.36	6.20	2.032	6.02 ^2^	6.59 ^1^	6.01 ^2^	6.60 ^1^	19.321 ***
	(1.87)	(1.77)		(1.86)	(1.84)		(1.84)	(1.81)	(1.77)	(1.89)	
**Internalization of social values**											
Self-transcendence											
Universalism	7.95	7.64	50.842 ***	7.86	7.73	0.475	7.73 ^2,a^	7.54 ^2^	7.37 ^2,b^	8.46 ^1^	99.959 ***
	(1.21)	(1.43)		(1.36)	(1.29)		(1.22)	(1.26)	(1.35)	(1.23)	
Benevolence	8.40	7.99	74.247 ***	8.18	8.23	2.432	7.95 ^3^	8.26 ^2^	8.04 ^3^	8.56 ^1^	37.326 ***
	(1.17)	(1.37)		(1.33)	(1.23)		(1.23)	(1.18)	(1.24)	(1.38)	
Conservation											
Security	7.85	7.65	15.907 ***	7.80	7.71	3.395	7.48 ^2,b^	7.58 ^2^	7.73 ^2,a^	8.20 ^1^	41.475 ***
	(1.34)	(1.41)		(1.36)	(1.4)		(1.30)	(1.29)	(1.38)	(1.41)	
Conformity	8.10	7.81	30.738 ***	7.98	7.93	0.093	7.76 ^2^	7.94 ^2,a^	7.67 ^2,b^	8.44 ^1^	46.350 ***
	(1.39)	(1.52)		(1.48)	(1.44)		(1.43)	(1.39)	(1.45)	(1.44)	
Tradition	6.78	6.73	3.610	6.81	6.70	0.067	6.41 ^3^	6.93 ^2^	6.11 ^4^	7.58 ^1^	117.692 ***
	(1.64)	(1.68)		(1.68)	(1.64)		(1.42)	(1.48)	(1.74)	(1.57)	

* *p* < 0.05; ** *p* < 0.01; *** *p* < 0.001; Bonferroni test: α = 0.05; 1 > 2 > 3 > 4; a > b.

**Table 4:** Means (and standard deviations) for parenting style, and the main univariate *F*-values for self-esteem and the internalization of social values (self-transcendence and conservation).

Socialization Outcomes	Parenting Style				
	Authoritative	Indulgent	Authoritarian	Neglectful	*F*(3, 2391)
**Internalization of social values**					
Self-transcendence					
Universalism	7.97 ^1^	8.11 ^1^	7.49 ^2^	7.64 ^2^	28.27 ***
	(1.21)	(1.23)	(1.45)	(1.34)	
Benevolence	8.39 ^1^	8.48 ^1^	7.87 ^3^	8.06 ^2^	27.14 ***
	(1.15)	(1.12)	(1.43)	(1.33)	
Conservation					
Security	8.03 ^1^	8.02 ^1^	7.45 ^2^	7.52 ^2^	31.05 ***
	(1.23)	(1.29)	(1.52)	(1.37)	
Conformity	8.23 ^1^	8.33 ^1^	7.51 ^3^	7.76 ^2^	43.71 ***
	(1.31)	(1.26)	(1.63)	(1.49)	
Tradition	6.95 ^1^	7.12 ^1^	6.45 ^2^	6.52 ^2^	24.51 ***
	(1.58)	(1.57)	(1.70)	(1.70)	

* *p* < 0.05; ** *p* < 0.01; *** *p* < 0.001; Bonferroni test: α = 0.05; 1 > 2 > 3 > 4.
